# Potential of ChatGPT to Pass the Japanese Medical and Healthcare Professional National Licenses: A Literature Review

**DOI:** 10.7759/cureus.66324

**Published:** 2024-08-06

**Authors:** Kai Ishida, Eisuke Hanada

**Affiliations:** 1 Faculty of Engineering, Shonan Institute of Technology, Fujisawa, JPN; 2 Faculty of Science and Engineering, Saga University, Saga, JPN

**Keywords:** medical education, academic performance, artificial intelligence, multimodal large language models, chatgpt

## Abstract

This systematic review aimed to assess the academic potential of ChatGPT (GPT-3.5, 4, and 4V) for Japanese national medical and healthcare licensing examinations, taking into account its strengths and limitations. Electronic databases such as PubMed/Medline, Google Scholar, and ICHUSHI (a Japanese medical article database) were systematically searched for relevant articles, particularly those published between January 1, 2022, and April 30, 2024. A formal narrative analysis was conducted by systematically arranging similarities and differences between individual research findings together. After rigorous screening, we reviewed 22 articles. Except for one article, all articles that evaluated GPT-4 showed that this tool could pass each exam containing text only. However, some studies also reported that, despite the possibility to pass, the results of GPT-4 were worse than those of the actual examinee. Moreover, the newest model GPT-4V insufficiently recognized images, thereby providing insufficient answers to questions that involved images and figures/tables. Therefore, their precision needs to be improved to obtain better results.

## Introduction and background

Recently, artificial intelligence (AI) has quickly gained popularity in various fields. Huge tasks that were solely performed by humans previously are now easily performed by AI-assisted software and robots expeditiously. In particular, multimodal large language models (MLLMs) can not only provide answers to questions but also generate new sentences, images, music, and videos. Released by OpenAI in November 2022, the Chat Generative Pretrained Transformer (ChatGPT) is an MLLM type that has attracted attention for its ability to generate detailed answers to questions in various fields [1]. GPT is a Transformer-based language model trained on a large corpus and produces text that resembles human speech. As of June 2024, the latest GPT-4 performance is in the top 10% of human examinees on the US bar exam [2]. Additionally, its performance on standardized tests in the United States is reportedly comparable to the average score of successful applicants to prestigious Ivy League universities. Moreover, a new model of ChatGPT, GPT-4V(ision), which was released in September 2023, has image recognition capability. Thus, more fields can utilize ChatGPT.

ChatGPT utilizes pretrained deep-learning algorithms from a huge amount of text data to generate human-like answers to questions entered in chat format. It includes abilities in reasoning, problem-solving, abstract thinking, and understanding complex ideas [3]. ChatGPT is designed to be a general-purpose conversational agent that can handle wide-ranging topics, thereby potentially useful for various areas, including customer service, chatbots, and education in various fields [4-6]. Additionally, it can possibly answer medical questions with a certain degree of accuracy [7,8]. In the medical field, ChatGPT can be applied to various areas. For example, it is being used to support the diagnosis of common complaints, screening of cancer, automatic generation of diagnostic reports, and applications in medical education [9-12]. Therefore, ChatGPT can assist medical and healthcare students and professionals. Its capability to pass various medical and healthcare licensing examinations has also been reported. Many countries have analyzed ChatGPT responses on the national examinations for physicians, pharmacists, and nurses [4,13-23].

In the use of MLLM, including ChatGPT, inaccuracies in professional content, biased responses, and erroneous information dissemination have been pointed out [5]. In education, some schools have published guidelines for using MLLM in report and paper writing. Additionally, since the COVID-19 outbreak that began at the end of 2019, more and more licensing examinations are being taken online, and MLLM can be used as a so-called cheating method. Therefore, when using MLLMs in education and examinations, the user's sense of ethics and the content of the generated data must be carefully examined.

The performance of MLLM varies in accuracy depending on the content of the questions and the field of study. Regarding the evaluation of answer accuracy for the national medical examination in the US and other countries, the GPT-4 generally obtains a passing level of knowledge [4,13-20], but in some languages, response accuracy was low [23]. Some studies systematically reviewed medical examinations that used GPT-3.5, but the results of examinations using the latest GPT-4/-4V have not yet been comprehensively analyzed [24,25]. Compared with its predecessor GPT-3.5, GPT-4 is reportedly “more reliable, creative, and able to handle many more nuanced instructions” [2]. OpenAI announced that GPT-4 could perform well in academic and specialized fields, with enhanced performance in languages other than English [2]. Furthermore, reviews focusing on specific languages ​​and encompassing national examinations for medical professions other than physicians remain unavailable. While ChatGPT is expected to become useful in medical education, the increasing reliance of students on it requires monitoring. Moreover, considering the application of GPT-4 to medical education and clinical practice in non-English-speaking countries, its reliability for clinical reasoning and medical knowledge in non-English languages should be confirmed. Therefore, evaluating the accuracy of ChatGPT responses is urgently required.

In this study, we aimed to conduct a systematic review of studies evaluating the accuracy of ChatGPT responses to national medical and healthcare licensing examinations in Japan. We also sought to identify the academic strengths and limitations of the ChatGPT on these examinations.

## Review

Method

All the procedures of the present systematic review were performed in accordance with the Preferred Reporting Items for Systematic Reviews and Meta-Analyses (PRISMA) guidelines. The review protocol registration in PROSPERO (International Prospective Register of Systematic Reviews), which requires that all the review processes follow the registration, was not applicable in this review.

Data Sources and Search Strategy

Electronic databases such as PubMed/Medline, Google Scholar, and ICHUSHI (a Japanese medical article database) were systematically and thoroughly searched for relevant articles, particularly those published between January 1, 2022, and April 30, 2024. Our search strategy mainly consisted of the following keywords combined with Medical Subject Headings terms and text words ((ChatGPT OR GPT-3.5 OR GPT-4)) AND ((Japan OR Japanese)) AND ((medical OR healthcare OR physician OR dentist OR pharmacist OR nurse OR therapist)) AND ((license OR license exam OR licensing exam OR national exam)). We included all the available and related articles in both English and Japanese languages. After de-duplication, we screened the titles of the acquired articles, followed by a full-text screening of the remaining articles.

Study Selection and Inclusion Criteria

We selected studies that met our predefined inclusion criteria as follows: (a) published as a scientific research paper or preprint; (b) written as a research paper or report, not a review, a meta-analysis, or a literature review; (c) conducted on ChatGPT; (d) targeted on Japanese national license examinations; and (e) evaluated its academic performance in any manner (marks obtained, whether passed or failed, etc.).

Conversely, the exclusion criteria were the following: articles that used AI platforms other than ChatGPT, examinations not related to medicine and/or healthcare, examinations that were not at a national level (e.g., specialist examinations certified by an academic society), and studies not mentioning the academic examination results given by ChatGPT.

Data Extraction

All the articles queried were exported to the EndNote Reference Library software (Clarivate Analytics). After a rigorous screening process, articles meeting the predefined inclusion criteria were selected. Desired data were extracted from each study by using a data extraction form. Table 1 shows the extracted information.

**Table 1:** Lists of extracted information

Lists of extracted information
Type, authors, and duration of the study
Type of national examination answered by ChatGPT
Type of GPT (GPT-3.5, 4, or 4V), input language (Japanese or English), whether or not the image was inputted
Type of analysis (question category, type, difficulty, academic field, whether or not images/tables were used, whether or not calculation was required, etc.)
Overall score
Key result
Academic limitations (automation bias, no insight, failure to interpret figures/tables, etc.)

Given that this study evaluated the accuracy of ChatGPT (GPT-3.5, 4, or 4V), we only concentrated on ChatGPT results for studies that compared MLLMs other than ChatGPT. For instance, if the study presented both performance results of ChatGPT and other MLLMs, but we analyzed ChatGPT (-3.5 or 4 or 4V) only.

Results

Literature Search

**Figure 1:**
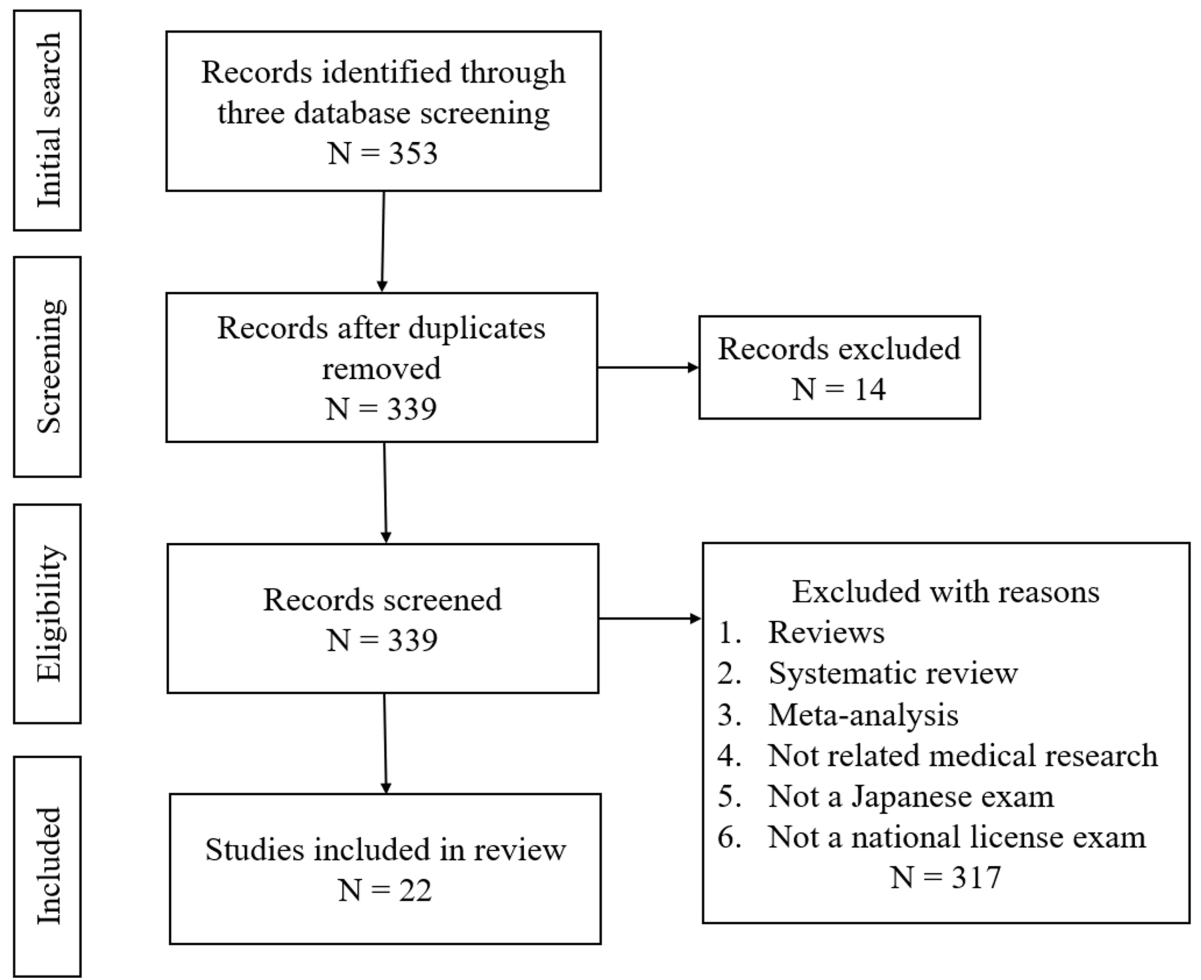
Flow diagram of the literature review

After the initial search, we selected 352 articles, and after removing duplicates and screening the titles/full texts, we included 22 articles for this review (Figure 1).

Study Characteristics

Table 2 shows the main characteristics of the 22 included studies [26-47]. Of these studies, 17 were written in English [26-34,36-42,46] (including two preprints [29,30]) and five in Japanese [35,43-44,47]. Regarding the target qualification examinations, we found nine papers for the Japanese National Medical License Examination (JNMLE) [26-34], three for Japanese National Dentist Examinations (JNDE) [35-37], two for Japanese National Exam for Pharmacists (JNEP) [38,39], two for Japanese National Nurse Exams (JNNE) [40,41], and one for each of the following: Japanese National Dental Hygienist Exam (JNDHE) [42], Japanese National Clinical Laboratory Technician Exam (JNCLTE) [43], Japanese National Physical Therapist Exam (JNPTE) [44], Japanese National Occupational Therapist Exam (JNOTE) [45], Japanese National Speech Therapist Exam (JNSTE) [46], and Japanese National Registered Dietitian Exam (JNRDE) [47]. Furthermore, 13 studies focused on single-year examinations only [26,27,30-33,36,37,39,41,42,46,47], and nine included multiple-year (two to six years) examinations [28,29,34,35,38,40,43-45].

**Table 2:** Main characteristics of the included studies

Study	Article type	Timing of implementation	Type of examination	Type of GPT	Input language	Image Input	Type of analysis	Result presentation
Yagita et al. [26]	Original article	January 2023	116th JNMLE	GPT-3.5/-4	Japanese	No	Question category and type	Correct answer and percentage
Kataoka et al. [27]	Short communication	February 2023	116th JNMLE	GPT-3.5/-4(Bing)	Japanese	No	Question category	Correct answer and percentage
Tanaka et al. [28]	Original article	February 2023	116th and 117th JNMLE	GPT-4	Japanese and English	No	Question category, type and each acadmic field	Mean
Kasai et al. [29]	Preprint	≦ March 2023	112th to 117th JNMLE	GPT-3/-4	Japanese	Yes	Question category	Number of correct answers
Kaneda et al. [30]	Preprint	March 2023	117th JNMLE	GPT-3.5	Japanese	No	Question type, whether or not have images	Number of correct answers
Takagi et al. [31]	Original article	March 2023	117th JNMLE	GPT-3.5/-4	Japanese	No	Question category, type and difficulty	Mean, Percentage with 95% confidence interval
Nakao et al. [32]	Original article	September 2023	117th JNMLE	GPT-4V	Japanese	Yes	Question type, whether or not images	Correct answer and percentage
Takagi et al. [33]	Letter	October 2023	117th JNMLE	GPT-4V	Japanese	Yes	Question category, type, whether or not images/tables	Mean, Percentage with 95% confidence interval
Kawahara et al. [34]	Original article	November 2023	112th to 117th JNMLE	GPT-4/-4V	Japanese	Yes	Question type, whether or not have images	Number of correct answers
Morishita et al. [35]	Original article (Japanese)	June to July 2023	112h to 115th JNDE	GPT-3.5/-4	Japanese	No	Question category, each academic field and number of specified correct answers	Number of correct answers
Ohta et al. [36]	Original article	≦ August 2023	116th JNDE	GPT-3.5/-4	Japanese	No	Question category	Number of correct answers
Morishita et al. [37]	Original article	October 2023	116th JNDE	GPT-4V	Japanese	Yes	Question category, each academic field, and number of specified correct answers	Number of correct answers
Kunitsu [38]	Original article	≦ April 2023	107th & 108th JNEP	GPT-4	Japanese	No	Question category, each academic field, whether or not requires calculation, whether the clinical case	Number of correct answers
Sato et al. [39]	Original article	November 2023	107th JNEP	GPT-3.5/-4V	Japanese	No	Each academic field and whether or not images/tables	Number of correct answers
Taira et al. [40]	Original article	≦ March 2023	108th to 112th JNNE	GPT-3.5	Japanese	No	Question category and each academic field	Number of correct answers
Kaneda et al. [41]	Original article	≦ July 2023	112th JNNE	GPT-3.5/ -4	Japanese	No	Question category	Number of correct answers
Yamaguchi et al. [42]	Original article	November 2023	32th JNDHE	GPT-3.5/-4	Japanese	No	Each academic field	Number of correct answers
Doi et al. [43]	Material (Japanese)	June to July 2023	67th to 69th JNCLTE	GPT-3.5/-4	Japanese	No	Each academic field	Number of correct answers
Sawamura et al. [44]	Short communication (Japanese)	June 2023	57th & 58th JNEPT	GPT-3.5/-4	Japanese	No	Question type	Number of correct answers
Kohiyama et al. [45]	Short communication (Japanese)	June 2023	57th & 58th JNEOT	GPT-3.5/-4	Japanese	No	Question type	Number of correct answers
Takeda et al. [46]	Original article	≦ February 2023	25th JNEST	GPT-3.5	Japanese	No	Type of multiple choice and each academic field	Number of correct answers
Kobayashi [47]	Original article (Japanese)	March 2023	37th JNRDE	GPT-4	Japanese	No	Each academic field	Number of correct answers

One study identified the prompt with the highest rate of correct answers in one year of testing [28] and then evaluated accuracy with optimized prompts in another year of testing. In many studies, a statement such as “You are a student taking a national exam; please indicate the correct answer according to the question text and images,” was used before asking students to solve the questions [27,34-37,40,42,44,45]. Meanwhile, no special prompt engineering was performed.

All but two papers [30,46] conducted validation using GPT-4/4V and/or by comparison with GPT-3.5. One study [27] compared the results of GPT-3.5 and Bing, which is based on GPT-4 but was treated as a GPT-4 research result. Five papers evaluated questions that included images and figures/tables using GPT-4V [32-34,37,39]. All studies were conducted after January 2023. The most recent ones were conducted in November 2023. Many studies presented data by subject area, such as essential, general, or specific disease. Furthermore, 10 studies included detailed analyses of performance by academic field [28,35,37-40,42,43,46,47], while some studies analyzed the images, charts, calculation problem presence/absence, and difficulty level [30-34,38,39].

ChatGPT's Overall Performance in National License Examinations

Table 3 presents the results of the systematic review. If multiple examinations were taken, the average score was used for the overall score. For studies without disclosure of the overall score, the available score was listed. Except for one study [35], all studies evaluating GPT-4 showed that this tool can pass each text-only exam. However, some reports claimed that despite the possibility of passing, its results were worse than those of the actual examinee. Among the studies evaluating GPT-3.5, some reported its ability to pass [46], but the majority showed that it did not reach the passing threshold.

**Table 3:** Results of the review

Study	Overall score		Key result	Academic limitation
	GPT-3.5	GPT-4/4V		
Yagita et al. [26]	42.80%	81.50%	GPT-4 has the potential as a diagnostic and therapeutic decision aid for physicians.	Only targeted questions without images.
Kataoka et al. [27]	38%	78%	The correct answer rate of GPT-4 was 78%. All incorrect answers in ChatGPT were attributed to “wrong information.”	Considering language is crucial when applying the LLM to other language translations.
Tanaka et al. [28]	―	Essential: 82.7% Basic & clinical: 77.2%	The best GPT-4 model with the optimized prompts scored 82.7% for the essential questions and 77.2% for the basic and clinical questions, both of which sufficed the minimum passing scoring rates of 80.0% and 74.6%, respectively.	Only targeted questions without images.
Kasai et al. [29]	41.80%	78.40%	The average score was about 30 points lower than the average score of actual examinees. There were also cases where contraindicated options were chosen.	LLMs sometimes select prohibited choices that should be strictly avoided in medical practice in Japan, such as suggesting euthanasia.
Kaneda et al. [30]	55%	―	GPT-3.5 did not reach the passing threshold.	ChatGPT due to room for improvement in performance.
Takagi et al. [31]	50.80%	79.90%	For difficult questions, GPT-4 had a higher correct answer rate than the actual examinee.	An absolute contraindication answers were not evaluated.
Nakao et al. [32]	―	input with image: 68% without image: 72%	The additional information from the images did not significantly improve the performance of GPT-4V in the exam.	Further analysis is necessary to determine whether its conclusions can be generalized to questions in other languages or of different types.
Takagi et al. [33]	―	78.20%	The correct response rate for questions with images was 71.9% for ChatGPT-4V.However, only 35% of questions that included tables were answered correctly.	Not considered the image quality. An absolute contraindication answers were not evaluated.
Kawahara et al. [34]	―	74.2%	GPT-4/-4V passed each exam that included images, illustrations, and pictures.	An absolute contraindication answers were not evaluated.
Morishita et al. [35]	42.20%	67.50%	There was a lack of knowledge in specific dental fields. In addition, the correct answer rate for questions that required multiple correct answers tended to be poor.	Only targeted questions without images. Reproducibility and variation of answers.
Ohta et al. [36]	51.90%	73.50%	GPT-4 was more accurate than GPT-3.5, but neither met the passing criteria for the required questions. Students performed significantly worse on questions in dentistry than in other fields.	Only targeted questions without images. Tested only once. The quality of GPT responses varies depending on the prompt.
Morishita et al. [37]	―	35%	The current evaluation of ChatGPT-4V’s image recognition capabilities revealed significant limitations in the context of the exam.	Analysis was conducted on questions from a single exam, and the results may be biased because of the small number of questions in each field.
Kunitsu [38]	―	63.70%	GPT-4 showed that some passing thresholds were not met in terms of the accuracy rate for all JNEP questions, but the accuracy rates for the questions that GPT-4 could answer met all of the passing thresholds.	Only targeted questions without images.
Sato et al. [39]	43.50%	72.50%	High accuracy rates were pharmacology and practice field. An accuracy rate of 36.1% for items that included diagrams.	Even within the same ChatGPT model, the accuracy may vary depending on the timing of the input test, owing to such updates.
Taira et al. [40]	Basic: 75.1% General: 64.5%	―	With additional learning, prompt engineering, and tuning of ChatGPT, it will likely exceed the passing threshold.	Only targeted questions without images. Not involve advanced prompt engineering
Kaneda et al. [41]	59.90%	79.70%	The correct answer rate was 90% in scenario-based questions in GPT-4.	Only targeted questions without images. Not scrutinize the basis of the answers. There is a possibility that the correct answer could be reached by chance. Detailed evaluation was not conducted.
Yamaguchi et al. [42]	63%	75.30%	The correct answer rate was 100% in some fields.	The evaluation was conducted only once. Only one exam was evaluated.
Doi et al. [43]	51.40%	79.80%	The 20% of incorrect answers included answers that could lead to misdiagnosis when diagnosing patients	Only targeted questions without images.
Sawamura et al. [44]	51.60%	77.10%	GPT-4 passed the exam, but GPT-3.5 did not reach the passing threshold.	Only targeted questions without images. The quality of GPT responses varies depending on the prompt. Possibility of AI hallucination.
Kohiyama et al. [45]	53.20%	78.40%	The correct answer rate for practical questions was higher than for general questions.	Only targeted questions without images.
Takeda et al. [46]	66%	―	GPT-3.5 reached the passing threshold and was slightly better than the actual examinee's performance.	Possibility of AI hallucination.
Kobayashi [47]	―	78.60%	The correct answer rate varied greatly depending on the field of question.	Only targeted questions without images.

In a study that evaluated the JNMLE using GPT-4, all but one paper [35] showed that the passing criteria were 80% or higher on required questions and 72% or higher on general questions, with the highest accuracy reaching 88.1% and 75.4%, respectively [26]. However, the average correct answer rate of the actual examinees for the 117th JMLE was 89.2% for required questions and 83.1% for general questions, and ChatGPT's performance was worse than this in both studies. However, the correct answer rate of GPT-4 for difficult questions was higher than that of the actual examinee. A limitation of these studies is that many questions did not include all questions and that questions with images or figures/tables were excluded or were evaluated without them being inputted. In some cases, ChatGPT candidates chose contraindicated options, such as recommending euthanasia, even though their abilities exceeded the passing threshold [29]. A study using GPT-4V that focused only on image questions reported that adding images did not improve performance compared with asking only text questions [32]. Additionally, of the papers evaluated using GPT-3.5, none reached the passing threshold [26,27,29-31].

Research on JNDE using GPT-4 has shown the possibility of passing the threshold, excluding questions with images and some areas [35,36]. In a study that focused on questions with images, GPT-4V had a lower correct answer rate than the actual examinee, making this tool unable to pass [37]. They also found a lack of knowledge about dentistry regardless of the presence or absence of images.

Moreover, two studies targeting JNEP using GPT-4 showed the possibility of passing the threshold [38,39]. One study in particular evaluated the accuracy of questions with images, and it passed the test. However, the correct answer rate for questions with figures and tables was low (36.1%) [39]. Additionally, in both studies, the correct answer rate was high in pharmacology, but the accuracy in physics and chemistry was poor.

In JNNE, GPT-4 obtained an extremely high score rate [41]. The correct answer rate was particularly high for conversation-style and scenario-based questions. Other studies have shown that in some years, GPT-3.5 was acceptable, but the overall score was low [42].

In JNEOT, GPT-4 achieved a correct answer rate of over 80% [45]. For practical questions, the correct answer rate was over 90%.

In other examinations, GPT-4 had a high score rate of 70%-80%, and GPT-3.5 had a low score rate of 40%-60% [42-44,46,47].

GPT-4V’s Overall Performance in Questions with Images or Figures/Tables

Three studies focusing on qualification examinations evaluated the accuracy of answers obtained using GPT-4V, which allows image input and covers all questions involving images and figures/tables but excludes inappropriate questions [33,34,39]. In addition, two studies focused only on image and diagram questions [32,37]. In these studies, the average correct answer rate for questions with images in JMLE was 60%-70% [32-34]. Additionally, the correct answer rate for questions with tables was only 35% [33]. A study of JNDE reported that among questions with images, the correct answer rate was 57.1% for required questions, 43.6% for general questions, 28.6% for clinical questions, and 35% overall [37]. In a study targeting JNEP, the correct answer rate for questions without figures/tables was 80%, whereas that for questions with figures/tables was low (36.1%) [39]. Therefore, the correct answer rate on ChatGPT generally decreases in questions that include images/tables or diagrams.

ChatGPT's Overall Performance in Each Academic Field

Ten papers were analyzed by the academic field of question [28,35,37-40,42,43,46,47]. A study of JNMLE using GPT-4, which had optimized prompts, reported a high rate of incorrect answers for public health and endocrinology questions [28]. In a study targeting JNDE, GPT-4 achieved a 100% accuracy rate in fields such as anesthesiology, radiology, and pharmacology [35]. However, ChatGPT reportedly lacks knowledge in areas such as dentures and conservative restorations. Additionally, in a study of image questions, the correct answer rate was relatively high for questions related to anesthesiology and endodontics, but the correct answer rate was 0% for questions related to anatomy, oral physiology, and oral pathology [37]. Two studies targeting JNEP reported good performance in pharmacology and pathophysiology but poor performance in physics and chemistry on GPT-4 [38, 39]. In JNNE, GPT-4 performed well in academic fields such as nutrition, pathology, hematology, ophthalmology, otorhinolaryngology, dentistry, and nursing practice. Conversely, it was poor in pharmacology, social welfare, law, endocrinology/metabolism, and dermatology [40]. In a study targeting JNDHE, the correct answer rate in the fields of disease mechanism, recovery process promotion, and the human body (excluding teeth and oral cavity) structure and function was 100% in GPT-4 [42]. For the theory of preventive dental procedures, the correct answer rate was low (54.5%). In a study of JNCLTE using GPT-4, general clinical laboratory medicine showed the highest rate, followed by medical engineering, hematology, microbiology, and physiology, with over 80% [43]. In contrast, public health was the worst, falling below 70%. In the JNSTE, GPT-3.5 obtained a correct answer rate of over 80% in fields such as basic medicine and clinical medicine; this percentage was better than that of the actual examinee [46]. However, the score for language development disorder and dysarthria (child) was below 50%, which was worse than that of the actual examinee. In a study targeting JNRDE, GPT-4 achieved a 100% correct answer rate for questions related to basic nutrition [47]. Moreover, the rates for public health and anatomy, physiology, and pathology exceeded 90%, whereas those for nutrition education theory and food and health were low.

Discussion

Overview of ChatGPT's Performance in Examinations for Japanese National Medical and Healthcare Professional Licensing

This review article targeted multiple national medical and healthcare examinations in Japan, and most of the reviewed studies reported that GPT-4 was above the minimum passing threshold. From the results of this review, GPT-4 showed superior performance compared with GPT-3.5 in all studies, possibly because GPT-4 learns more text data than GPT-3.5 as a result of an increase in the number of parameters, leading to improved higher capability [48]. However, in many cases, the accuracy was less than the actual examinee score. In particular, recognition accuracy was poor for questions involving images and figures/tables. Currently, the correct answer rate for these questions is not excellent because of the level of accuracy for image recognition and ChatGPT's own knowledge. Additionally, response accuracy tended to be poor for questions with calculations or for questions requiring multiple correct answers [35,37,38]. However, in the future, as image recognition accuracy, computational power, and problem comprehension improve, the rate of correct answers may increase.

ChatGPT output changes depending on the content of the input prompt [49]. In one paper targeting GPT-4, by adjusting the prompt using the previous year's questions and performing input after tuning such as translating from Japanese to English, this tool successfully met the minimum passing threshold of JNMLE [28]. Conversely, a paper that comprehensively reviewed the results of English medical examinations reported that GPT-3.5 could possibly pass most of the examinations taken without any special prompt adjustments [25]. In many of the reviewed studies that used input in Japanese, GPT-3.5 insufficiently passed several Japanese national medical and healthcare licensing examinations. However, in the evaluation using GPT-4, almost all studies met the passing threshold even without special prompt adjustments or translation from Japanese to English. As mentioned above, the performance of GPT-4 is considerably improved compared with GPT-3.5, and even without special prompt engineering, it can pass Japanese national medical and healthcare examinations. Thus, the recognition accuracy for languages other than English has also improved. In fact, OpenAI reported a slight difference in performance related to language differences in GPT-4 [48]. Regarding the characteristics of the questions asked, in a study that evaluated JNEOT, the correct answer rate was higher for practical questions than for general questions [45]. In JNNE, the correct answer rate for scenario-based questions was high [41]. These questions contain detailed patient information and medical conditions. From a prompt engineering perspective, ChatGPT may have a high ability to handle problems with detailed condition settings such as this.

As pointed out in several studies, even if we ask the same question to ChatGPT, the answer may be different each time, and providing a unique answer to the same question is not necessarily possible [35]. Therefore, the answers obtained should be carefully examined. Furthermore, many studies have questioned the existence of hallucination in the commentary generated by ChatGPT [5]. One reason for this existence is that the original training data contain errors and biases, which may result in incorrect information [50,51]. Therefore, several studies have pointed out the possibility of ChatGPT to provide answers that can lead to misdiagnosis or unethical judgment [29,43]. Thus, presently, even GPT-4 must be used with caution for medical purposes. We need to check the accuracy of the generated answers and verify the information.

Limitations of This Study

First, this review cannot evaluate input in languages other than English and Japanese. However, as mentioned above, in GPT-4, the difference in performance related to language differences was only slight [48], and in fact, even in languages other than English and Japanese, it can possibly pass the national medical licensing examinations [16-19]. Second, this study did not cover all national medical and healthcare licensing examinations, including those for radiology technicians, clinical engineers, and emergency medical technicians, conducted in Japan. Basic medical topics such as anatomy, physiology, pathology, pharmacology, and public health are commonly asked in healthcare qualifications. However, even in the same academic field, the difficulty level of the questions varies depending on the qualification. In fact, ChatGPT showed a relatively good performance for public health in JNRDE [47] but was poor in JNMLE and JNCLTE [28,43]. In addition, a bias exists in the organs and diseases that each qualification specializes in. For example, speech therapists are required to have knowledge of ears and throat, whereas physical therapists are required to have knowledge of muscles and bones. Furthermore, the specialized fields covered vary depending on the qualification. For instance, radiology technicians are required to have knowledge of radiation physics and radiation biology, whereas clinical engineers are required to have knowledge of medical device safety management and biological function substitution. The learning content of ChatGPT is also influenced by each academic field. Moreover, in the questions asked at JNRDE, the Japanese unique way of thinking about cooking and the existence of standards and systems compiled by the Japanese government may be the main reasons for incorrect answers on ChatGPT [47]. The explanation is that, in other countries, the correct answer rate is low for questions requiring country-specific knowledge and that learning is likely to be insufficient [18]. Furthermore, given that the accuracy in recognizing images and figures/tables is insufficient, tests, wherein these frequently appear, may not achieve sufficient response accuracy. Therefore, performance can vary depending on each qualification test because of these factors. However, considering that candidates are expected to have some knowledge of general basic medicine and clinical medicine, which are commonly asked questions, GPT-4 will meet the passing criteria for many examinations.

Finally, although this study comprehensively reviewed the accuracy analysis of ChatGPT for Japanese national medical and healthcare licensing examinations, it did not examine the direct educational effects of ChatGPT. Many studies reported that ChatGPT has a higher possibility of passing the national licensing exam, but no reports specifically evaluated the educational effects according to the research results, indicating another limitation of the study. However, by comprehensively reviewing ChatGPT's answer accuracy, this study was able to evaluate whether ChatGPT can indeed generate appropriate answers. In the future, this study could become the basis for verifying the effectiveness of education using ChatGPT. Future research should explore more deeply the impact of education using ChatGPT on the acquisition of knowledge in the specialized area of each qualification.

## Conclusions

ChatGPT (GPT-4/4V) passed many national medical and healthcare licensing examinations in Japan, obtaining a considerably better performance than the previous version GPT-3.5. However, in many cases, although they met the minimum threshold for passing, they performed worse than the actual examinee. Additionally, the accuracy of recognizing images and charts is currently an issue. If these precisions were improved, the results would be even better.
